# Association of Subthalamic Deep Brain Stimulation With Motor, Functional, and Pharmacologic Outcomes in Patients With Monogenic Parkinson Disease: A Systematic Review and Meta-analysis

**DOI:** 10.1001/jamanetworkopen.2018.7800

**Published:** 2019-02-01

**Authors:** Carlo Alberto Artusi, Alok K. Dwivedi, Alberto Romagnolo, Gian Pal, Marcelo Kauffman, Ignacio Mata, Dhiren Patel, Joaquin A. Vizcarra, Andrew Duker, Luca Marsili, Binith Cheeran, Daniel Woo, Maria Fiorella Contarino, Leonard Verhagen, Leonardo Lopiano, Alberto J. Espay, Alfonso Fasano, Aristide Merola

**Affiliations:** 1 Department of Neuroscience Rita Levi Montalcini, University of Torino, Torino, Italy; 2 Texas Tech University Health Sciences Center El Paso, El Paso; 3 Rush University Medical Center, Chicago, Illinois; 4 Consultorio de Neurogenética-Centro Universitario de Neurologia y Division Neurologia-Hospital J. M. Ramos Mejia-CONICET, Buenos Aires, Argentina; 5 Genomic Medicine, Lerner Research Institute, Cleveland Clinic, Cleveland, Ohio; 6 Gardner Family Center for Parkinson's Disease and Movement Disorders, Department of Neurology, University of Cincinnati, Cincinnati, Ohio; 7 Abbott Laboratories, Austin, Texas; 8 The London Clinic, London, United Kingdom; 9 Department of Neurology, Leiden University Medical Center, Leiden, the Netherlands; 10 Department of Neurology, Haga Teaching Hospital, The Hague, the Netherlands; 11 Morton and Gloria Shulman Movement Disorders Clinic, Edmond J. Safra Program in Parkinson’s Disease, Toronto Western Hospital, University Health Network, Toronto, Ontario, Canada; 12 Division of Neurology, University of Toronto, Toronto, Ontario, Canada; 13 Krembil Research Institute, Toronto, Ontario, Canada

## Abstract

**Importance:**

Comparative outcomes among different monogenic forms of Parkinson disease after subthalamic nucleus deep brain stimulation (STN DBS) remain unclear.

**Objective:**

To compare clinical outcomes in patients with the most common monogenic forms of Parkinson disease treated with STN DBS.

**Design, Setting, and Participants:**

Systematic review and meta-analysis in which a PubMed search of interventional and noninterventional studies of Parkinson disease with* LRRK2, GBA,* or *PRKN* gene mutations published between January 1, 1990, and May 1, 2018, was conducted. Among the inclusion criteria were articles that reported the Motor subscale of the Unified Parkinson’s Disease Rating Scale Part III (UPDRS-III) before and after STN DBS treatment, that involved human participants, and that were published in the English language. Studies that used aggregated data from patients with different genetic mutations were excluded, and so were studies with assumed but not confirmed genetic data or incomplete follow-up data.

**Main Outcomes and Measures:**

Changes in UPDRS-III scores and levodopa equivalent daily dose (LEDD) were analyzed for each monogenic form of Parkinson disease. Additional end points included activities of daily living (UPDRS-II), motor complications (UPDRS-IV), and cognitive function.

**Results:**

Of the 611 eligible studies, 17 (2.8%) met the full inclusion criteria; these 17 studies consisted of 8 cohort studies (47.1%), 3 case series (17.6%), and 6 case reports (35.3%), and they involved a total of 518 patients. The UPDRS-III score improved by 46% in *LRRK2* (mean change, 23.0 points; 95% CI, 15.2-30.8; *P* < .001), 49% in *GBA* (20.0 points; 95% CI, 4.5-35.5; *P* = .01), 43% in *PRKN* (24.1 points; 95% CI, 12.4-35.9; *P* < .001), and 53% in idiopathic Parkinson disease (25.2 points; 95% CI, 21.3-29.2; *P* < .001). The LEDD was reduced by 61% in *LRRK2* (mean change, 711.9 mg/d; 95% CI, 491.8-932.0; *P* < .001), 22% in *GBA* (269.2 mg/d; 95% CI, 226.8-311.5; *P* < .001), 61% in *PRKN* (494.8 mg/d; 95% CI, –18.1 to –1007.8; *P* = .06), and 55% in idiopathic Parkinson disease (681.8 mg/d; 95% CI, 544.4-819.1; *P* < .001). Carriers of the *PRKN* mutations showed sustained improvements in UPDRS-II and UPDRS-IV, whereas *LRRK2* mutation carriers sustained improvements only in UPDRS-IV. Carriers of the *GBA* mutation showed worse postsurgical cognitive and functional performance.

**Conclusions and Relevance:**

Treatment with STN DBS for patients with Parkinson disease with* LRRK2, GBA,* or *PRKN* mutations appears to be associated with similar motor outcomes but different changes in dopaminergic dose, activities of daily living, motor complications, and cognitive functions.

## Key Points

**Question:**

Does the outcome of subthalamic deep brain stimulation vary among common monogenic forms of Parkinson disease?

**Findings:**

In this systematic review and meta-analysis involving 518 patients from 17 published studies, treatment with subthalamic deep brain stimulation for patients with Parkinson disease with *LRRK2*, *GBA*, or *PRKN* gene mutation yielded similar motor outcomes but different changes in dopaminergic doses, activities of daily living, motor complications, and cognitive functions.

**Meaning:**

Genetic screening for *LRRK2, GBA,* and *PRKN* mutations in patients with Parkinson disease who are candidates for subthalamic deep brain stimulation may serve to inform outcomes.

## Teaser

This systematic review and meta-analysis examines research articles indexed in PubMed on the genetic mutations in Parkinson disease and their response to subthalamic nucleus deep brain stimulation treatment.

## Introduction

The traditional view of Parkinson disease as a single idiopathic disorder has been useful for the development of symptomatic treatments, such as dopaminergic oral medications. However, the selection of optimal candidates for subthalamic nucleus deep brain stimulation (STN DBS) demands a more nuanced characterization of the distinctive and heterogeneous pathogenic mechanisms involved in the different subtypes of Parkinson disease.

A range of genetic mutations has been associated with variable clinical phenotypes of Parkinson disease. Carriers of glucosylceramidase β (*GBA *[OMIM *606463]) gene mutations, for instance, have a greater probability of developing cognitive impairment, postural instability, and falls.^1^ Carriers of *PRKN *(OMIM *602544), *PINK1 *(OMIM *608309), and *DJ-1* (OMIM *602533) gene mutations, on the contrary, exhibit milder progression of motor and nonmotor features.^2^

Treatment with STN DBS can yield greater than 50% of motor improvement,^3,4,5^ 60% amelioration of levodopa-related motor complications,^4,5^ 40% to 60% improvement in quality of life,^6,7^ and 50% reduction in the levodopa equivalent daily dose (LEDD).^8^ Nonetheless, the clinical outcomes after STN DBS have remained variable,^9^ with nearly half of patients with Parkinson disease developing stimulation-resistant symptoms such as gait impairment, postural instability, falls, cognitive impairment, and other nonmotor deficits within 5 years from the procedure.^9^ This variability in outcomes warrants an examination of clinical and biologic factors. To this end, we sought to examine whether different monogenic forms of Parkinson disease are associated with different responses to STN DBS in motor, functional, and pharmacologic end points.

## Methods

This study followed the Preferred Reporting Items for Systematic Reviews and Meta-analyses (PRISMA) reporting guideline and the Meta-analysis of Observational Studies in Epidemiology (MOOSE) guideline.^10,11^

### Search Methods

We searched PubMed for interventional and noninterventional studies published between January 1, 1990, and May 1, 2018, that reported data on patients treated with STN DBS and screened for monogenic forms of Parkinson disease. We used the following search terms: *deep brain stimulation, mutation, gene, genetics, inherited, familial, Parkinson's disease, *and *parkinsonism*.

Three of us (C.A.A., A.R., and D.P.) independently reviewed abstracts and full-text articles for eligibility criteria. Duplicated studies were identified and excluded. Only studies that referred to human participants and were published in the English language were considered. No restrictions were applied to participant sex, age, ethnicity, follow-up duration, disease duration, or disease severity. The reference list of each article was screened for additional pertinent studies not captured by the original search strategy.

### Inclusion and Exclusion Criteria

We included studies in which patients with genetically confirmed monogenic forms of Parkinson disease were treated with STN DBS, that included a minimum postsurgical follow-up of 3 months, and that reported the Motor subscale of the Unified Parkinson’s Disease Rating Scale Part III (UPDRS-III) in the presurgical medication-off and postsurgical medication-off/stimulation-on conditions.^12^ Studies of aggregated data from patients with different genetic mutations (eg, genetic data were pooled rather than reported separately) were excluded. We excluded studies with assumed but not confirmed genetic data or incomplete follow-up data. For recessive mutations such as *PRKN*, only data from homozygous or compound heterozygous were included. Data from heterozygous carriers were extracted but only evaluated in ancillary analyses.

### Study End Points

We used a data collection form to extract the following variables of interest: (1) UPDRS-III in the presurgical medication-off and postsurgical medication-off/stimulation-on conditions; (2) LEDD, according to a previously published conversion table^13^; (3) UPDRS Part II (activities of daily living); (4) UPDRS Part IV (motor complications); and (5) cognitive evaluation by Montreal Cognitive Assessment,^14^ Mini–Mental State Examination,^15^ or Mattis Dementia Rating Scale,^16^ according to availability. Additional data included in the data collection were study population, sample size, genetic mutations evaluated, year of publication, study design, age at Parkinson disease onset, disease duration at STN DBS, and follow-up duration in months.

Data were expressed as mean with SD or mean percentage change, as appropriate. If multiple data points were available from the same cohort, we included the most recent publication with the longest follow-up. The control group was formed by patients with Parkinson disease from the same study data sets with confirmed negative genetic screening.

### Assessment of Risk of Bias

Two of us (C.A.A. and D.P.) independently performed the quality appraisal of qualifying studies. Given the heterogeneity of study designs, the risk of bias of individual studies was evaluated using the National Heart, Lung, and Blood Institute quality appraisal tools, per the Cochrane handbook recommendations.^17^ Visual inspection of funnel plots was conducted to assess for publication bias.^18^

### Statistical Analysis

Two sets of analyses were performed: (1) meta-analyses for quantitative outcomes (UPDRS-III and LEDD) from different studies with varying sample sizes after assigning appropriate weights by specific gene mutations and controls, and (2) descriptive data analyses without weights for case report studies (intraindividual analyses) and outcomes of variable definitions and measurements, such as activities of daily living, motor complications, and cognitive outcomes. In the descriptive data analyses, we used summary statistics (mean, SD, and range) for continuous data and frequency for categorical data. To estimate the proportion of specific genetic mutations, we conducted for each gene a separate meta-analysis for proportions, using a random-effects model with the DerSimonian and Laird method.^19^

The 95% CI for proportion was computed according to the score (Wilson) method. Mean percentage change in study outcomes were converted to mean and SD wherever feasible. The effect size for each end point was computed using the mean change between the presurgical and postsurgical periods along with the pooled SD. After estimating the correlation coefficients between presurgical and postsurgical values for a specific gene mutation, pooled SD was computed for each data set using a previously published formula.^20^ The heterogeneity in the studies was measured with the *I*^2^ statistic, which provides the proportion of observed variance likely to remain even after eliminating sampling error.^21^ An *I*^2^ statistic greater than 50% was considered as substantial heterogeneity.^17^ Given the sample size, inclusion of observational studies, and heterogeneity across the studies, the pooled effect size was computed using a random-effects model by means of the DerSimonian and Laird method.^19^ We further confirmed the findings of the study by performing Hartung-Knapp-Sidik-Jonkman method for a random-effects meta-analysis.^22^

Given the small number of studies included, we performed validation analyses with 2 different methods to estimate the pooled SD and confirm the robustness of the meta-analysis–estimated associations between the presurgical and postsurgical treatment for each specific gene. In the validation analysis, meta-analyses using a random-effects model with the DerSimonian and Laird method were conducted for each outcome using the pooled SD, computed after estimating the correlation coefficient between presurgical and postsurgical values for all data sets (irrespective of gene mutations) and ignoring the correlation between presurgical and postsurgical values. Publication bias was assessed using the Egger test and a funnel plot. Key findings were displayed using forest plots.

Two-sided *P* < .05 was considered statistically significant. Data sets for meta-analyses and statistical codes were included (eTables 1 to 5 and eAppendix 1 in the Supplement). Our biostatistician (A.D.) carried out the analyses using Stata, version 13.1 (StataCorp LLC) (eAppendix 2 in the Supplement).

## Results

**Figure 1.**
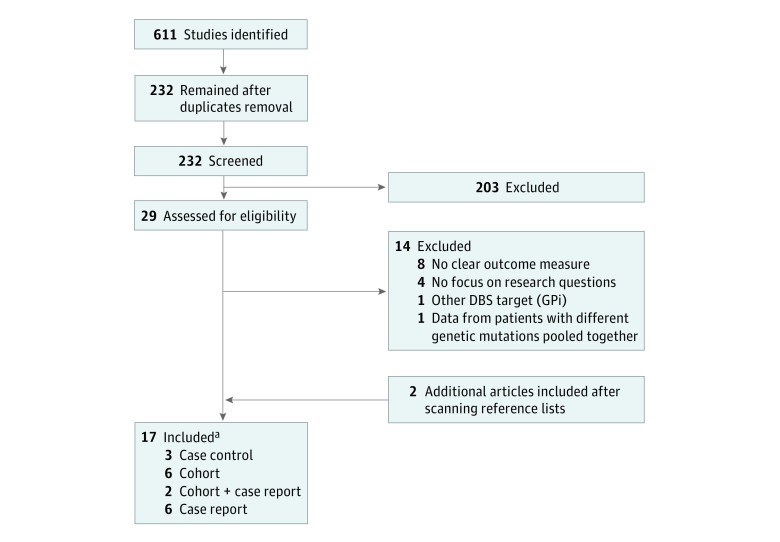
Study Flowchart DBS indicates deep brain stimulation; GPi, globus pallidus pars interna. ^a^Twenty-four patients were carriers of heterozygous *PRKN* mutation and were analyzed separately.^23,24,25,26,28,29,30^

Of the 611 eligible studies, 17 (2.8%) met the full inclusion criteria (8 cohort studies [47.1%], 3 case series [17.6%], and 6 case reports [35.3%])^23,24,25,26,27,28,29,30,31,32,33,34,35,36,37,38,39^ and underwent data extraction, individual quality assessment, and risk-of-bias evaluation (Figure 1). Of the 17 studies, 9 (53.0%) yielded data sets for meta-analysis, 6 (35.3%) for intraindividual analysis, and 2 (11.8%) for both. No signs of publication bias were detected through visual inspection of funnel plots and publication bias tests.

### Clinical and Demographic Data

We found a total of 518 patients (135 with monogenic forms of Parkinson disease and 383 controls) from 17 studies of Parkinson disease–associated genetic mutations (Table 1). Twelve carriers (8.9%) of monogenic Parkinson disease were excluded because of non-STN targeting (n = 9)^24^ or incomplete follow-up data (n = 3).^26,33^ Five controls (1.3%) were excluded because of incomplete follow-up data.^25,26^

**Table 1.** Reviewed Studies

Source	Study Design	Patients Screened, No.	Selection Criteria	Patients Genetically Assessed, No.	Gene	Patients With Genetic Mutation, No.	Patients for Meta-analysis (Motor), No.	Patients for Meta-analysis (Therapy), No.	Control Patients, No.	Mean Follow-up, mo	Mean (SD), y				Quality Assessment
											Age at PD Onset in Carriers	PD Duration at DBS in Carriers	Age at PD Onset in Controls	PD Duration at DBS in Controls	
**Studies Included in Meta-analysis**															
Romito et al,^28^ 2005	Cohort	36	NA	36	*PRKN*	1 *PRKN* ^a^; 4 heterozygous *PRKN*	4 Heterozygous *PRKN*	4 Heterozygous *PRKN*	31	18	*PRKN *heterozygous: 33.0 (5.8)	*PRKN *heterozygous: 15.7 (4.9)	43.5 (6.2)	13.9 (5.5)	Fair
Schüpbach et al,^31^ 2007	Cohort	69	NA	69	*LRRK2*	9 Heterozygous *LRRK2*	9 Heterozygous *LRRK2*	9 heterozygous *LRRK2*	60	12	41.1 (6.1)	13.4 (2.7)	43.1 (7.8)	13.0 (8.2)	Fair
Gómez-Esteban et al,^33^ 2008	Cohort	48	Family history	8	*LRRK2*	5 Heterozygous *LRRK2*^b^	4 Heterozygous *LRRK2*	NA	43	6	43.2 (10.8)	12.8 (3.6)	58.0 (1.2)	14.2 (6.9)	Fair
Moro et al,^23^ 2008	Cohort	312	Age at PD onset <45 y	80	*PRKN* *PINK1* *LRRK2*	6 *PRKN*; 5 heterozygous *PRKN*; 1 homozygous *PINK1*^a^	6 *PRKN*; 5 heterozygous *PRKN*	NA	68	12	*PRKN*: 26.5 (10.1); *PRKN* heterozygous: 34.4 (6.4)	*PRKN*: 22.2 (8.1); *PRKN* heterozygous: 15.6 (3.6)	36.3 (7.0)	17.3 (7.4)	Fair
Lohmann et al,^25^ 2008	Cohort	134	Young onset and/or family history	54	*PRKN* *LRRK2*	7 *PRKN*; 7 heterozygous *PRKN*	7 *PRKN*; 7 heterozygous *PRKN*	7 *PRKN*; 7 heterozygous *PRKN*	40 (1 excluded from meta-analysis)	18	*PRKN*: 26.4 (9.6); *PRKN *heterozygous: 38.4 (9.9)	*PRKN*: 19.9 (7.9); *PRKN* heterozygous: 13.4 (2.2)	38.0 (9.2)	15.0 (4.6)	Fair
Weiss et al,^36^ 2012	Case-control	98	*GBA* mutation	98	*GBA*	3 Heterozygous *GBA*	3 Heterozygous *GBA*	NA	6^c^	24-48	49.7 (3.8)	17.3 (5.5)	49.3 (5.1)	17.8 (6.8)	Fair
Angeli et al,^24^ 2013	Cohort	94	NA	94	*PRKN* *PINK1* *LRRK2* *DJ1* *SNCA* *GBA*	5 *PRKN* (2 STN DBS) 3 heterozygous *PRKN* (DBS target NR); 5 heterozygous *LRRK2* (5 STN DBS); 16 heterozygous *GBA* (13 STN DBS)^d^	2 *PRKN*; 5 heterozygous *LRRK2* 13 heterozygous *GBA*	2 *PRKN*; 5 heterozygous *LRRK2*; 13 heterozygous *GBA*	67	12	*PRKN*: 39.7 (1.2) *PRKN *heterozygous: NR; *LRRK2*: 43.0 (8.7); *GBA *heterozygous: 42.9 (6.2)	*PRKN*: 25.2 (12.8); *PRKN* heterozygous: NR *LRRK2*: 12.1 (1.8); *GBA* heterozygous: 11.2 (5.0)	40.8 (7.2)	15.1 (5.5)	Fair
Greenbaum et al,^32^ 2013	Case-control	NR	*LRRK2* mutation	NR	*PRKN* *PINK1* *LRRK2*	13 Heterozygous *LRRK2*	13 Heterozygous *LRRK2*	13 Heterozygous *LRRK2*	26^e^	12	49.5 (6.8)	11.7 (4.9)	49.2 (6.6)	13.2 (5.8)	Good
Kim et al,^26^ 2014	Cohort	122	Age at PD onset <40 y	18	*PRKN* *PINK1* *LRRK2* *DJ1* *SNCA*	3 *PRKN*; 2 heterozygous *PRKN*^f^	3 *PRKN*	3 *PRKN*	13 (4 Excluded from meta-analysis)	45	*PRKN*: 21.7 (8.5); *PRKN* heterozygous: NR	*PRKN*: 28.3 (7.6); *PRKN* heterozygous: NR	34.6 (3.9)	15.4 (3.4)	Good
Sayad et al,^29^ 2016	Cohort	27	NA	27	*PRKN* *PINK1* *LRRK2* *DJ1*	2 Heterozygous *PRKN*; 15 heterozygous *LRRK2*	2 Heterozygous *PRKN*; 15 heterozygous *LRRK2*	NA	12 (Authors included 2 hetorzygous *PRKN* patients as controls)	24	*PRKN *heterozygous: 48.0 (0.0); *LRRK2*: 40.1 (9.4)	*PRKN *heterozygous: 11.5 (2.1); *LRRK2*: 16.1 (3.0)	40.3 (8.2)	14.3 (2.7)	Good
Lythe et al,^37^ 2017	Case-control	NR	*GBA* mutation	NR	*PRKN* *PINK1* *LRRK2* *DJ1* *SNCA* *GBA*	15 Heterozygous *GBA*; 2 homozygous *GBA*	15 Heterozygous *GBA*; 2 homozygous *GBA*	15 Heterozygous *GBA*; 2 homozygous *GBA*	17^g^	90	41.4 (5.8)	12.1 (1.3)	43.0 (5.3)	14.7 (5.0)	Good
Total patients included in meta-analysis, No.	NA	NA	NA	NA	NA	NA	115	80	378	NA	NA	NA			
**Case Reports (Intraindividual Patient Analysis)**															
Capecci et al,^27^ 2004	Case report	NA	NA	1	*PRKN*	1 *PRKN*	NA	NA	NA	12	22	19	NA	NA	NA
Romito et al,^28^ 2005	Case report	36	NA	36	*PRKN*	1 *PRKN*	NA	NA	NA	18	45	8	NA	NA	NA
Moro et al,^23^ 2008	Case report	312	Age at PD onset <45 y	80	*PRKN* *PINK1* *LRRK2*	1 Homozygous *PINK1*	NA	NA	NA	12	31	30	NA	NA	NA
Breit et al,^34^ 2010	Case report	NA	NA	1	*LRRK2*	1 Heterozygous *LRRK2*	NA	NA	NA	12	42	18	NA	NA	NA
Stefani et al,^35^ 2013	Case report	NA	NA	1	*LRRK2*	1 Heterozygous *LRRK2*	NA	NA	NA	3	49	7	NA	NA	NA
Antonini et al,^38^ 2012	Case report	NA	NA	1	*SNCA*	1 Heterozygous *SNCA*	NA	NA	NA	12	41	5	NA	NA	NA
Nakahara et al,^39^ 2014	Case report	NA	NA	1	*PRKN* *PINK1*	1 *PRKN *+ heterozygous *PINK1*	NA	NA	NA	8	15	45	NA	NA	NA
Genç et al,^30^ 2016	Case report	NA	NA	1	*PRKN*	1 Heterozygous *PRKN*	NA	NA	NA	NR	10	4	NA	NA	NA

Abbreviations: DBS, deep brain stimulation; NA, not applicable; NR, not reported; PD, Parkinson disease; *PRKN*, homozygous or compound heterozygous *PRKN* mutations; STN, subthalamic nucleus.

^a^ Data reported in the Case Reports section.

^b^ One patient was excluded from meta-analysis because of incomplete data.

^c^ Control group matched 1:2 with patients with *GBA* mutation (sex, age, PD duration at STN DBS).

^d^ Nine patients were excluded from meta-analysis because of DBS target other than STN or unknown.

^e^ Control group matched 1:2 with patients with *LRRK2* mutation (sex, age at onset, PD duration at STN DBS).

^f^ Two patients were excluded from meta-analysis because of incomplete data.

^g^ Control group matched 1:1 with patients with *GBA* mutation (sex, PD duration at STN DBS).

In the population tested for specific mutations, the proportion of *LRRK2* carriers was 29% (95% CI, 10%-47%)^24,29,31,32,33^; *GBA *carriers, 5.0% (95% CI, 2%-8%)^24,36,37^; and *PRKN *carriers, 6.0% (95% CI, 2%-10%) (Table 2).^23,24,25,26,28^ Our search yielded only 1 case of *PINK1*,^23^ 1 case of *SNCA *(OMIM *163890),^38^ and 1 case of combined *PRKN* and *PINK1* mutations (Table 3).^39^ The proportion of heterozygous *PRKN* carriers was 9.0% (95% CI, 5%-12%) (eTable 6 in the Supplement).^23,24,25,26,28,29,30^

**Table 2.** Proportion and Type of Mutations

Gene	Patients With Mutation, % (95% CI)	Source	Gene Assessment	Type of Mutation Found
*PRKN*	6 (2-10)	Capecci et al,^27^ 2004	*PRKN*	ex3del
		Romito et al,^28^ 2005	*PRKN*	G828A-duplEx1
		Moro et al,^23^ 2008	*PRKN*; *PINK1*; *LRRK2 *(only G2019S)	Q34fsX43 (2 patients); N58_Q178del; V2445fsX318; Q57fsX96-Q347fsX368; I2fsX7-Q311fsX318
		Lohmann et al,^25^ 2008	*PRKN*; *LRRK2* (only G2019S)	C289G; ex5del–255delA; ex3del–prom-ex1del; ex2-4dupl–ex3del; ex5del–C441R; ex2del–ex3del; ex4-7del–IVS7-1G>C
		Angeli et al,^24^ 2013	*PRKN*; *PINK1*; *LRRK2 *(exons 1, 2, 10, 15, 27, 41, 49); *DJ-1* (exons 3, 5, 6, 7); *SNCA*; *GBA*	c.101_102delAG; ex3-4del; c.1289G>A-c.823C>T; c.337_376del-c.465–466del; c.823C>T-ex6dupl
		Kim et al,^26^ 2014	*PRKN*; *PINK1*; *LRRK2 *(only G2019S); *DJ-1*; *SNCA*	NR (3 patients)
*LRRK2*	29 (10-47)	Schüpbach et al,^31^ 2007	*LRRK2 *(exon 41)	G2019S (8 patients); T2031S
		Gómez-Esteban et al,^33^ 2008	*LRRK2*	R1441G (5 patients)
		Breit et al,^34^ 2010	*LRRK2*	R793M
		Stefani et al,^35^ 2013	*LRRK2* (exon 41)	G2019S
		Angeli et al,^24^ 2013	*PRKN*; *PINK1*; *LRRK2* (exons 1, 2, 10, 15, 27, 41, 49); *DJ-1* (exons 3, 5, 6, 7); *SNCA*; *GBA*; *PRKN*; *PINK1*; *LRRK2 *(only G2019S); *PRKN*	G2019S (5 patients)
		Greenbaum et al,^32^ 2013	*PINK1*; *LRRK2* (exon 41); *DJ-1*; *GBA *(N370S and L444P)	G2019S (13 patients)
		Sayad et al,^29^ 2016	*PRKN*	G2019S (15 patients)
*GBA*	5 (2-8)	Weiss et al,^36^ 2012	*PINK1*; *LRRK2 *(exons 1, 2, 10, 15, 27, 41, 49); *DJ-1* (exons 3, 5, 6, 7); *SNCA*; *GBA*; *PRKN*	L444P (2 patients); N370S

		Angeli et al,^24^ 2013	*PINK1*; *LRRK2*; *DJ1*; *SNCA*; *GBA*; *PRKN*	13 Patients heterozygous: E326K (4 patients); N370S; D409H; recNcil; R463C; N188S; R275Q; IVS211 G>A; L444P; T369M 3 patients homozygous/compound heterozygous: E326K; R463C; L444P-E326K
		Lythe et al,^37^ 2017	*PINK1*; *LRRK2 *(only G2019S); *SNCA*	15 Patients heterozygous: NR; 2 patients homozygous: NR
*PINK1*	NA	Moro et al,^23^ 2008	*PRKN*	V170G
*SNCA*	NA	Antonini et al,^38^ 2012	*PINK1*	dupl 4q22.1
*PRKN + PINK1*	NA	Nakahara et al,^39^ 2014	NA	T175PfsX2 (*PRKN*) + R58-V59insGR (heterozygous *PINK1*)

Abbreviations: NA, not applicable; NR, not reported.

**Table 3.** Intraindividual Patient Analyses

Genes	No. of Studies	Mean (Range)		Improvement (Range), %
		Baseline Values	Improvement	
Motor improvement (UPDRS-III score)^a^				
* PRKN*	2	53 (45-61)	32 (26-38)	64 (43-94)
* LRRK2*	2	48.5 (27-70)	32.5 (19-46)	68 (66-70)
* PINK1*	1	35.5	16.5	47
* SNCA*	1	22	9.5	43
* PRKN + PINK1*	1	86	53	62
LEDD reduction, mg				
* PRKN*	2	700 (500-900)	406 (220-592)	55 (44-66)
* LRRK2*	2	875 (850-900)	445 (400-490)	51 (44-58)
* PINK1*	0	NA	NA	NA
* SNCA*	1	1250	790	63
* PRKN + PINK1*	1	1181	691	59

Abbreviations: LEDD, levodopa equivalent daily dose; NA, not applicable; UPDRS-III, Unified Parkinson’s Disease Rating Scale Part III.

^a^ Motor improvement was defined as the change in the UPDRS-III score between the presurgical medication-off condition and the postsurgical medication-off/stimulation-on condition. The presurgical motor outcome (UPDRS-III score) associated with levodopa is reported in eTable 9 in the Supplement.

The mean (SD) age at Parkinson disease onset was 43.4 (3.7) years in *LRRK2*, 44.7 (4.4) years in *GBA*, and 28.6 (7.7) years in *PRKN *carriers. For the single cases, the age at onset was 31 years in *PINK1*, 41 years in *SNCA*, and 15 years in the combined *PRKN *and *PINK1 *carriers. The mean (SD) age at onset in the control population was 44.6 (6.7) years. The mean (SD) disease duration at the time of STN DBS was 13.4 (1.6) years in *LRRK2*, 13.5 (3.3) years in *GBA*, and 23.9 (3.6) years in *PRKN* carriers. For the single cases, it was 30 years in the *PINK1*, 5 years in *SNCA*, and 45 years in the combined *PRKN* and *PINK1 *carriers. The mean (SD) disease duration at the time of STN DBS among the controls was 14.6 (1.4) years.

### Meta-analysis

#### Motor End Points

Of the 123 patients with Parkinson disease–associated genetic mutations, 115 (93.5%; 46 *LRRK2*, 33 *GBA,* 18 homozygous *PRKN,* and 18 heterozygous *PRKN*) were included in the meta-analysis for motor end points, and 8 single cases (6.5%) underwent intraindividual patient analyses (Tables 1 and 3).

**Figure 2.**
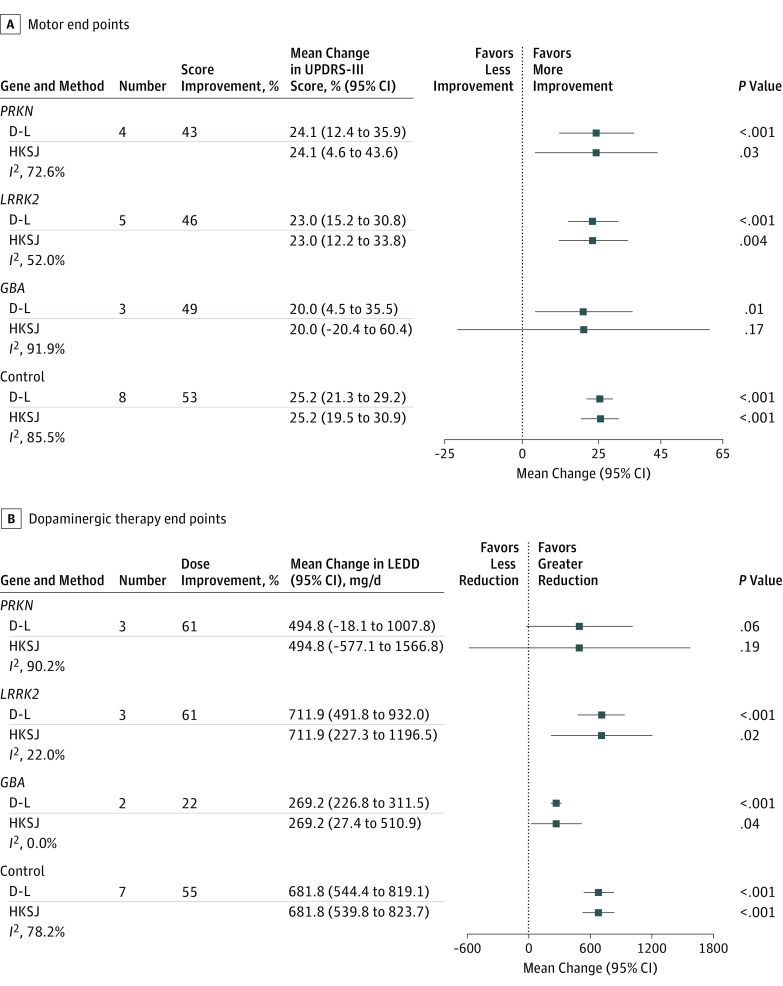
Meta-analysis of Motor Improvement and Levodopa Equivalent Daily Dose (LEDD) Reduction After Subthalamic Nucleus Deep Brain Stimulation A, The DerSimonian and Laird (D-L) meta-analysis method produced slightly less precise estimates compared with the Hartung-Knapp-Sidik-Jonkman (HKSJ) method. Both methods produced similar findings, except for the *GBA* gene owing to the extremely high heterogeneity and small number of studies. The presurgical motor outcome (Unified Parkinson’s Disease Rating Scale Part III [UPDRS-III] score) associated with levodopa is reported in eTable 8 in the Supplement. B, Both D-L and HKSJ meta-analysis methods produced similar findings for all genes. Error bars represent the 95% CI of the mean changes reported.

The UPDRS-III score improved by 46% in *LRRK2* (mean change, 23.0 points; 95% CI, 15.2-30.8; *P* < .001), 49% in *GBA* (20.0 points; 95% CI, 4.5-35.5; *P* = .01), 43% in *PRKN* (24.1 points; 95% CI, 12.4-35.9; *P* < .001), and 53% in control (25.2 points; 95% CI, 21.3-29.2; *P* < .001) patients (Figure 2A and eFigure in the Supplement). Data from the heterozygous *PRKN* carriers are reported in eTable 6 in the Supplement. The validation analyses confirmed the robustness of the findings (eTable 7 in the Supplement).

#### Dopaminergic Therapy

Among the 123 patients with Parkinson disease–associated genetic mutations, 80 (65.0%) (27 *LRRK2*, 30 *GBA*, 12 homozygous *PRKN*, and 11 heterozygous *PRKN*) were included in the meta-analysis for therapy end points, and 7 single cases (5.7%) underwent intraindividual patient analyses (Tables 1 and 3).

The LEDD was reduced by 61% (mean change, 711.9 mg/d; 95% CI, 491.8-932.0 mg/d; *P* < .001) in *LRRK2*, 22% (269.2 mg/d; 95% CI, 226.8-311.5 mg/d; *P* < .001) in *GBA*, 61% (494.8 mg/d; 95% CI, −18.1 to 1007.8 mg/d; *P* = .06) in *PRKN*, and 55% (681.8 mg/d; 95% CI, 544.4-819.1 mg/d; *P* < .001) in control patients (Figure 2B and eFigure in the Supplement). Data from the heterozygous *PRKN* carriers are reported in eTable 6 in the Supplement. The validation analyses confirmed the robustness of the findings (eTable 7 in the Supplement). The presurgical motor outcome (UPDRS-III score) associated with levodopa is reported in eTables 8 and 9 in the Supplement.

### Systematic Review of Individual-Level Data

#### Activities of Daily Living

Three studies and 5 case reports analyzed changes in the UPDRS-II in patients with a monogenic form of Parkinson disease treated with STN DBS.^26,27,31,33,34,35,38,39^ The *LRRK2* carriers (n = 15) (4 studies with a mean [SD] follow-up of 14.4 [22.5] months) showed variable results, varying from 45.2% to 66.7% improvement in the G2019S variant (n = 10) (2 studies with a mean [SD] follow-up of 11.1 [2.8] months)^31,35^ and 21.4% in the R793M variant (n = 1) (1 study with a 12-month follow-up)^34^ to 10.0% deterioration in the R1441G variant (n = 4) (1 study with a 6-month follow-up).^33^ The *PRKN* carriers (n = 4) (2 studies with a mean [SD] follow-up of 36.8 [16.5] months) showed improvement by 62.0% to 81.8%.^26,27^ The single case of *SNCA* showed a 37.2% improvement at 12-months of follow-up, and the single case of combined *PRKN* and *PINK1* showed a 12.5% worsening at 8 months of follow-up.^38,39^

#### Motor Complications

Five studies and 4 case reports involved motor complications (UPDRS-IV) in patients with a monogenic form of Parkinson disease treated with STN DBS.^24,25,26,27,31,34,36,38,39^ Improvements were observed in *LRRK2* carriers (n = 15) (3 studies with a mean [SD] follow-up of 12.0 [0.0] months) by 50% to 75%,^24,31,34^ *GBA* carriers (n = 16) (2 studies with a mean [SD] follow-up of 14.3 [4.8] months) by 37% to 80%,^24,36^ and *PRKN* carriers (n = 13) (4 studies with a mean [SD] follow-up of 22.8 [12.9] months) by 20% to 100%.^24,25,26,27^ The single case of *SNCA* improved by 87.5% at 12 months, and the combined* PRKN* and *PINK1* case improved by 80% at 8 months.^38,39^

#### Cognitive Outcomes

Six studies and 1 case report analyzed cognitive data in patients with a monogenic form of Parkinson disease treated with STN DBS.^24,25,28,31,36,37,38^ The *LRRK2* carriers (n = 9) (1 study with 12-month follow-up) had stable postsurgical Mattis Dementia Rating Scale scores.^31^ The *GBA* carriers (n = 26) (3 studies with a mean [SD] follow-up of 72.2 [21.1] months) developed progressive cognitive decline after STN DBS.^24,36,37^ A 7-year follow-up study found worse performance in all of the 5 cognitive domains in 17 *GBA* carriers, compared with 17 controls.^37^ A 5-year prospective study found a steeper decline in Mattis Dementia Rating Scale scores in 13 *GBA* carriers, compared with 67 controls,^24^ and a case series of 3 *GBA* carriers and 6 controls found a higher prevalence of dementia in the *GBA* group after 24 to 48 months of STN DBS.^36^ The *PRKN* carriers (n = 8) (2 studies with a mean [SD] follow-up of 18.0 [0.0] months) showed no or minimal postsurgical cognitive decline in the Mattis Dementia Rating Scale score^25^ and at full neuropsychological testing.^28^ Finally, the single case of *SNCA* (1 study with a follow-up of 12 months) showed a 1-point loss in the Mini–Mental State Examination (from 30 to 29 points).^38^

## Discussion

The results of this systematic review and meta-analysis confirmed that STN DBS is consistently associated with improved motor outcomes in monogenic forms of Parkinson disease. However, STN DBS showed differences in LEDD reduction, motor complications, and cognitive outcomes.

The overall proportion of monogenic Parkinson disease carriers in this meta-analysis (2%-8% for *GBA*, 2%-10% for *PRKN*, and 10%-47% for *LRRK2*) was in keeping with findings in previous studies, suggesting a relatively high prevalence of *LRRK2*, *GBA*, and *PRKN* mutations in Parkinson disease cohorts selected for surgical treatments.^40^ Although this observation highlights the importance of clarifying the contribution of genetic factors to functional outcomes after STN DBS, the variability associated with monogenic forms of Parkinson disease (eg, the age at onset ranged from 15 years in combined* PRKN *and* PINK1* carriers to 44 years in *GBA* carriers; the duration of disease at STN DBS varied from 5 years in *SNCA* carriers to 45 years in combined *PRKN *and* PINK1* carriers) and the associated but unmeasured epigenetic factors should be considered when interpreting these results.

The *LRRK2* mutation in Parkinson disease showed an excellent motor response to STN DBS, with 46% reduction in the UPDRS-III score and more than 60% reduction in dopaminergic therapy. Carriers of the G2019S, the most frequent variant in the *LRRK2* gene, had activities-of-daily-living outcomes similar to those of carriers of idiopathic Parkinson disease, whereas carriers of the R1441G variant rapidly deteriorated after STN DBS.^33^ These findings are in agreement with the notion that G2019S-associated Parkinson disease exhibits a milder motor decline and slower progression of medication- and stimulation-resistant symptoms compared with idiopathic Parkinson disease.^41,42^ So far, 7 missense mutations have been identified within the *LRRK2* gene, accounting for 1% to 2% of all cases of Parkinson disease.^43^ The G2019S variant is by far the most prevalent, whereas 6 other variants are infrequently observed, with the exception of the R1441G variant in patients of Basque descent.^44^ No clear differences have been identified in the phenotypes associated with these mutations, but rarer mutations seem to have higher clinical penetrance.^45^ Still, current data remain insufficient to definitively conclude that G2019S variant carriers receive a more favorable outcome after STN DBS.

The *GBA *mutation in Parkinson disease exhibited a substantial improvement of motor symptoms but a considerably higher rate of cognitive complications compared with other monogenic forms of Parkinson disease, and lower LEDD reduction after STN DBS (22% vs 55% of patients with sporadic Parkinson disease).^24,36,37^

We cannot exclude that the knowledge that *GBA* mutation leads to a more aggressive clinical phenotype^46^ could affect the therapeutic decision of maintaining higher post-DBS doses of dopaminergic medications. Carriers of the *GBA* mutation may present with a spectrum of clinical phenotypes, from akinetic-rigid Parkinson disease to dementia with Lewy bodies, with variable motor complications in the form of dyskinesia and wearing-off. In a cohort of 20 patients with the *GBA *mutation in Parkinson disease, compared with 27 patients with sporadic Parkinson disease, the mutation was found to be associated with a relatively younger age at onset and more rapid progression of cognitive symptoms, postural instability, and gait abnormalities.^46^ Depression, anxiety, social dysfunction, and hallucinations may also be more frequently observed in these patients.^47,48,49,50^ Up to 5% of patients with Parkinson disease undergoing STN DBS might be carriers of the *GBA* mutation, but we could not clarify which of the numerous *GBA* variants is the most represented in this specific population.^51^ Overall, this meta-analysis confirms the motor advantages of STN DBS for *GBA* mutation carriers but suggests a higher rate of cognitive complications. A thorough neuropsychological assessment and a careful discussion of the risk/reward profile of STN DBS are, therefore, important in this particular population. Future studies will need to examine whether DBS of the globus pallidus pars interna should be preferred by *GBA* mutation carriers, given the possible lower rate of cognitive complications.^52^

The *PRKN *mutation in Parkinson disease showed a good response to STN DBS, with substantial improvement of motor complications and a relatively low prevalence of dementia up to 4 years after surgical treatment. These data suggest that this population might be particularly suitable for STN DBS, particularly because of the early development of dyskinesia and other levodopa-related motor fluctuations.^53^ On the other hand, the high prevalence of behavioral and psychiatric symptoms among *PRKN* mutation carriers warrants a careful neuropsychological evaluation before these carriers’ eligibility for STN DBS is considered.^53,54^ The *PRKN* mutation is the most common known cause of early-onset Parkinson disease, accounting for up to 77% of familial Parkinson disease with an age at onset younger than 30 years^55^ and 10% to 20% of early-onset Parkinson disease in general.^56^ Approximately 30% of *PRKN* mutations result from single-nucleotide polymorphism changes, 10% from small deletions, and more than 50% from deletions or duplications of 1 or several exons.^57^

Heterozygous *PRKN* mutations are not deemed pathogenic, but the possibility exists that cases of homozygous or compound heterozygous *PRKN* mutation have been erroneously diagnosed in older studies as heterozygous, given that not all exons were tested or gene doses analyses performed. Results from this selected subgroup showed a 41% motor improvement after STN DBS, compared with 53% in the control group, and a 76% LEDD reduction, compared with 55% controls (eTable 6 in the Supplement). In sum, these data suggest that STN DBS in carriers of *PRKN* mutations (both homozygous and heterozygous) might yield motor improvements at least comparable to what has been observed in patients with sporadic or idiopathic Parkinson disease.

Only single cases reported the outcomes of STN DBS in rarer forms of monogenic Parkinson disease. Single case reports showed moderate motor improvements in *SNCA* and *PINK1 *mutations, as well as in a case with combined *PRKN* and* PINK1 *mutation. Still, the variability in the pattern of progression associated with these rare genetic variants renders these data of uncertain value at this time. Carriers of the *SNCA* mutation are prone to developing cognitive decline, autonomic dysfunction, speech problems, and behavioral changes, which may affect the overall outcome of STN DBS.^58^ Carriers of the *PINK1* mutation, on the other hand, usually manifest a slow progression of nonmotor symptoms,^59^ which suggests that this particular subtype of Parkinson disease may be a good candidate for STN DBS to address motor complications. However, the high prevalence of psychiatric symptoms has to be considered in the presurgical screening.^60^

No data have been reported for carriers of the *DJ-1* mutation, a rare autosomal recessive monogenic form of early-onset Parkinson disease. Mutations in the *DJ-1*, *PRKN,* and *PINK1* genes might present with a similar phenotype, characterized by an age at onset of 25 to 30 years, mild nonmotor symptoms, and a tendency to develop dyskinesia and dystonia in response to even minimal doses of levodopa, which may be optimally treated with STN DBS.^42^

This study suggests that *LRRK2, GBA,* and *PRKN* mutations in Parkinson disease are associated with motor improvements after STN DBS, comparable to idiopathic Parkinson disease. Carriers of the G2019 variant in *LRRK2* and *PRKN* mutations showed sustained advantages in motor complications and activities of daily living, whereas *GBA* mutation carriers frequently developed cognitive impairment and stimulation-resistant symptoms within 2 to 7 years after surgical treatment. Whether this latter finding resulted from an incomplete response to STN DBS or to a faster accrual of disability intrinsic to the *GBA* phenotype remains unclear. The limited data available for *SNCA* and *PINK1* mutations highlight the critical unmet need for large, multicenter studies aimed at characterizing the natural pattern of disease progression associated with rare genetic variants of Parkinson disease. Emerging possibilities for a more common genetic analysis arise from a substantial drop in the cost (and therefore wider availability) of gene panels that are designed to detect the presence of pathogenic variants in *SNCA*, *LRRK2*, *PRKN*, *GBA*, *PINK1*, *DJ-1*, and *VPS35* (OMIM *601501) genes. Considering the pathogenic role of copy number variations in the pathogenesis of *SNCA* and *PRNK* mutations in Parkinson disease, these gene panel assays should combine sequencing and gene doses. Exome sequencing might be a more comprehensive alternative to predesigned gene panels, but difficulties remain in thoroughly assessing *PRKN* and *GBA* gene variability.^61^

### Limitations

Several limitations should temper the strength of these results. First, the vast heterogeneity of the sample size, demographic features, and outcome measures of the source studies prevented the possibility of conducting a meta-analysis of the association between STN DBS and motor complications, activities of daily living, and cognitive outcomes. The analysis of these data was, therefore, limited to a systematic review of the few cases reported in the literature. Second, the sample size of the monogenic variants examined is low, consistent with their low prevalence but also with inconsistent genotyping across clinics. Third, the prevalence of the genetic mutations in Parkinson disease may have been overestimated because of a selection bias toward genetically screening patients with early-onset Parkinson disease or with a family history of neurodegenerative disorders. Variability in disease duration and length of observation period may also have accounted for some of the differences observed.

Fourth, the differences in study designs and length of follow-up, among other variables, generated large heterogeneity across the eligible studies. The unknown frequency of monogenic Parkinson disease variants precluded sensitivity analyses, but validation analyses confirmed the consistency of our results. Fifth, the cosegregation of factors known to be associated with clinical outcomes after STN DBS, such as young age at onset and at surgical treatment as well as the usually younger age of patients with monogenic forms of Parkinson disease, might have played a role in the observed results.^8,62,63^ Because the predictive interval for the estimated pooled effect could not be reported owing to the small number of studies, the extent to which the differences in clinical outcomes might be associated with a heterogeneous genetic background or with factors that cosegregate with the genetic background needs to be clarified in large multicenter prospective clinical trials.

## Conclusions

This meta-analysis and systematic review suggests that patients with Parkinson disease who are carriers of *LRRK2*,* GBA*, and* PRKN* gene mutations show good motor advantages after STN DBS, comparable to patients with idiopathic Parkinson disease. Patients with the G2019 variant of *LRRK2* and *PRKN* mutations showed sustained advantages on motor complications and activities of daily living, whereas patients with *GBA* mutations frequently developed cognitive impairment and stimulation-resistant symptoms within 2 to 7 years after surgical treatment. However, the current level of evidence remains insufficient to recommend genetic screening in patients with Parkinson disease who are considered candidates for STN DBS. Larger, ideally prospective, studies may establish the areas in which genetic information serves to inform the process of selecting the optimal candidates for advanced therapy for Parkinson disease.

## Notes

Supplement. **eFigure.** Percent Motor Improvement and Dopaminergic Dose Reduction Following STN DBS **eTable 1.** Meta-analysis Data for Proportion **eTable 2.** Meta-analysis Data for Motor and Therapy Endpoints **eTable 3.** Case Report Data **eTable 4.** Meta-analysis Output **eTable 5.** Motor Levodopa Response **eTable 6.** Heterozygous *PRKN* Analysis **eTable 7.** Validation analyses for UPDRS-III improvement and LEDD reduction **eTable 8.** Pre STN DBS Motor Outcome (UPDRS-III Score) Associated With Levodopa - Meta-analysis **eTable 9.** Pre STN DBS Motor Outcome (UPDRS-III Score) Associated With Levodopa - Intra-Individual Patient Analysis **eAppendix 1.** Statistical Codes **eAppendix 2.** Methods of Data Analysis
