# Supplementary material for: Association of Subthalamic Deep Brain Stimulation With Motor, Functional, and Pharmacologic Outcomes in Patients With Monogenic Parkinson Disease: A Systematic Review and Meta-analysis
Source: JAMA Netw Open. 2019 Feb 1;2(2):e187800. doi: 10.1001/jamanetworkopen.2018.7800 (PMC6484599; doi:10.1001/jamanetworkopen.2018.7800)
Supplement: Supplement. — eFigure. Percent Motor Improvement and Dopaminergic Dose Reduction Following STN DBS eTable 1. Meta-analysis Data for Proportion eTable 2. Meta-analysis Data for Motor and Therapy Endpoints eTable 3. Case Report Data eTable 4. Meta-analysis Output eTable 5. Motor Levodopa Response eTable 6. Heterozygous PRKN Analysis eTable 7. Validation analyses for UPDRS-III improvement and LEDD reduction eTable 8. Pre STN DBS Motor Outcome (UPDRS-III Score) Associated With Levodopa - Meta-analysis eTable 9. Pre STN DBS Motor Outcome (UPDRS-III Score) Associated With Levodopa - Intra-Individual Patient Analysis eAppendix 1. Statistical Codes eAppendix 2. Methods of Data Analysis [file jamanetwopen-2-e187800-s001.pdf]

Supplementary Online Content

Artusi CA, Dwivedi AK, Romagnolo A, et al. Association of subthalamic deep brain stimulation with motor, functional, and pharmacologic outcomes in patients with monogenic Parkinson disease: a systematic review and meta-analysis. *JAMA Netw Open.* 2019;2(2):e187800. doi: 10.1001/jamanetworkopen.2018.7800

eFigure. Percent Motor Improvement and Dopaminergic Dose Reduction Following STN DBS

eTable 1. Meta-analysis Data for Proportion

eTable 2. Meta-analysis Data for Motor and Therapy Endpoints

eTable 3. Case Report Data

eTable 4. Meta-analysis Output

eTable 5. Motor Levodopa Response

eTable 6. Heterozygous *PRKN* Analysis

eTable 7. Validation Analyses for UPDRS-III Improvement and LEDD Reduction

eTable 8. Pre STN DBS Motor Outcome (UPDRS-III Score) Associated With Levodopa - Meta-analysis

eTable 9. Pre STN DBS Motor Outcome (UPDRS-III Score) Associated With Levodopa - Intra-individual Patient Analysis

eAppendix 1. Statistical Codes

eAppendix 2. Methods of Data Analysis

This supplementary material has been provided by the authors to give readers additional information about their work.

©2019 Artusi CA et al. *JAMA Network Open.*

eFigure. Percent motor improvement and dopaminergic dose reduction following STN DBS

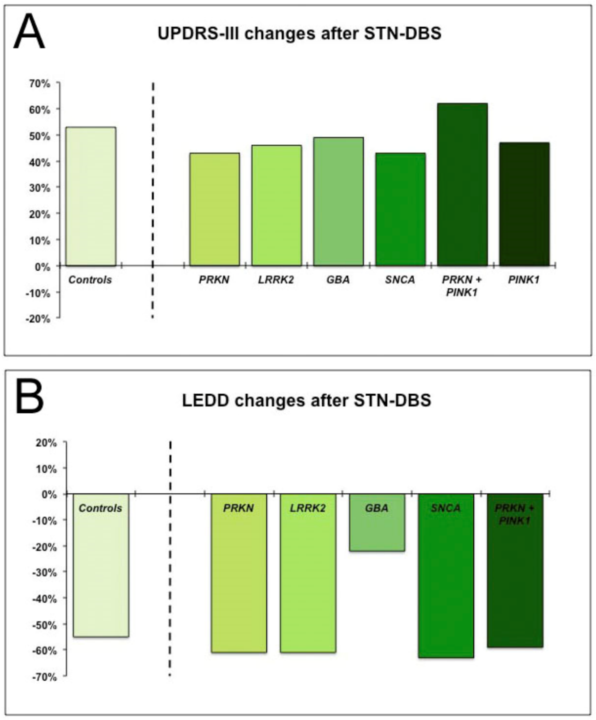

UPDRS: Unified Parkinson's Disease Rating Scale; LEDD: Levodopa Equivalent Daily Dose
STN-DBS: Subthalamic nucleus Deep brain stimulation
LEDD: Levodopa Equivalent Daily Dose

Motor improvement (A), defined as the percentage change in the motor subscale (part III) of the Unified Parkinson's Disease Rating Scale between the pre-surgical Medication-OFF condition and the post-surgical Medication-OFF/Stimulation-ON condition.
Changes of dopaminergic dose (B), defined as the modification of LEDD between the pre-surgical assessment and the post-surgical follow-up.

©2019 Artusi CA et al. *JAMA Network Open.*

eTable 1. Meta-analysis data for proportion

ID study	Authors	Year	Study	Total sample size	Screened	Mutation	*Parkin*	*Parkin hetero-zygous*	*LRRK2*	*PINK1*	*GBA*
**1**	Moro et al	2008	Cohort	312	80	12	6	5		1	
**2**	Angeli et al	2013	Cohort	94	93	19	2		5		13
**3**	Romito et al	2005	Cohort	36	36	5	1	4			
**4**	Lohmann et al	2008	Cohort	134	54	14	7	7			
**6**	Kim et al	2014	Cohort	122	18	5	3	2			
**7**	Schupbach et al	2007	Cohort	69	69	9			9		
**8**	Greenbaum et al	2013	Case-Control	39							
**9**	Sayad et al	2016	Cohort	27	27	17		2	15		
**11**	Lythe et al	2017	Case-Control	34							
**12**	Gomez-Esteban et al	2006	Case-Control	48	8	5			5		
**13**	Weiss et al	2012	Case-Control	98	98	3					3

©2019 Artusi CA et al. *JAMA Network Open.*

eTable 2A. Meta-analysis data for motor and therapy endpoints

ID study	Study	Unique study	Year	Study type	Genes	Gene type	Target	Total sample size	Age at onset	Disease duration at DBS	Follow-up months	Total genes
**1**	Moro et al (a)	1	2008	Cohort	PARKIN (homozygous)	1	STN	80	26.5	22.17	12	6
**2**	Moro et al (b)		2008	Cohort	PARKIN (heterozygous)	2	STN	80	34.4	15.6	12	5
**3**	Angeli et al (a)	1	2013	Cohort	PARKIN (homozygous), GBA, LRRK2	1	STN, Gpi, Vim	94	39.67	25.2	12	2
**4**	Romito et al	1	2005	Cohort	PARKIN (heterozygous)	2	STN	36	33	15.7	18	4
**5**	Lohmann et al (a)	1	2008	Cohort	PARKIN (homozygous)	1	STN	53	26.4	19.9	18	7
**6**	Lohmann et al (b)		2008	Cohort	PARKIN (heterozygous)	2	STN	53	38.4	13.4	18	7
**7**	Kim et al	1	2014	Cohort	PARKIN (homozygous)	1	STN	12	21.7	28.3	45	3
**8**	Schupbach et al	1	2007	Cohort	LRRK2	3	STN	69	41.1	13.4	12	9
**9**	Greenbaum et al	1	2013	Case-Control	LRRK2	3	STN	39	49.5	11.7	12	13
**10**	Sayad et al (a)	1	2016	Cohort	LRRK2	3	STN	27	40.1	16.1	24	15
**11**	Sayad et al (b)		2016	Cohort	PARKIN (heterozygous)	2	STN	27	48	11.5	24	2
**12**	Lythe et al	1	2017	Case-Control	GBA	4	STN, Gpi	34	41.4	12.1	90	17
**13**	Angeli et al (b)		2013	Cohort	PARKIN (homozygous), GBA, LRRK2	3	STN, Gpi, Vim	94	43	12.8	12	5
**14**	Angeli et al (c)		2013	Cohort	PARKIN (homozygous), GBA, LRRK2	4	STN, Gpi, Vim	94	42.9	11.2	12	16
**15**	Gomez-Esteban et al	1	2006	Case-control	LRRK2	3	STN	8	43.2	12.8	6	4

©2019 Artusi CA et al. *JAMA Network Open.*

**16**	Weiss et al	1	201 2	Case-Control	GBA	4	STN	98	49.7	17.33	36	3

STN: subthalamic nucleus
GPi: globus pallidus pars interna
Vim: nucleus ventralis intermedius
DBS: deep brain stimulation

©2019 Artusi CA et al. *JAMA Network Open.*

eTable 2B. Meta-analysis data for motor and therapy endpoints

ID study	Male mutated	Female mutated	Number of mutated patients	Presurgical UPDRS-III mutated	Presurgical UPDRS-III SD mutated	Postsurgical UPDRS-III mutated	Postsurgical UPDRS-III SD mutated	Number of controls	Male controls	Female controls	Age at onset controls	Disease duration controls	Presurgical UPDRS-III controls	Presurgical UPDRS-III SD controls	Postsurgical UPDRS-III controls	Postsurgical UPDRS-III SD controls
**1**	3	3	6	49.2	13.4	36.2	15.6	68								
**2**	2	3	5	50	9.1	30.7	7.9	68			3.3	1.3	48.7	13.1	21.5	10.7
**3**			2	62.5	3.5	43	0	67	46	21	40.8	15.1				
**4**	3	1	4	57	10.55	22.75	9.6	31	17	14	43.5	13.9	59.7	11,3	29	12.3
**5**			7	55.4	17.3	14.5	12.5	39								
**6**			7	54.3	13.9	17.8	11.2	39					51.9	18.3	17.9	15.1
**7**	1	2	3	49.8	24.5	24.7	14	9	5	4	34.6	15.4	38.3	10.6	17.2	5.5
**8**	6	3	9	41.4	12.4	17.8	9.6	60	34	26	43.1	13	43.4	17	15.7	9.9
**9**	8	5	13	42.5	11.8	28.5	13.1	26	17	9	49.2	13.2	43.3	12.3	27.2	14.1
**10**			15	55.8	16.4	27.3	20.6	12			40.3	14.3	51.7	14.4	38.5	16.6
**11**	2		2	47.5	2.1	51	0	12								
**12**	10	0	17	52.4	13	18.4	14.9	17	10	7	43	14.7	40.5	12	12.6	7.4
**13**	3	2	5	65.4	14.9	30.6	16.1	67								
**14**	9	7	13	50.5	12.4	28	11.4	67								
**15**			4	48.5	18.5	39.7	39.7	43			58	14.2				
**16**	0	3	3	18.7	4.6	17.3	11.7	6	0	6	49.3	17.8			18.3	7.1

UPDRS-III: unified parkinson’s disease rating scale part III
SD: standard deviation

©2019 Artusi CA et al. *JAMA Network Open.*

eTable 2C. Meta-analysis data for motor and therapy endpoints

ID study	Presurgical LEDD mutated	Presurgical LEDD SD mutated	Postsurgical LEDD mutated	Postsurgical LEDD SD mutated	Mean changed LEDD	SD changed LEDD	Presurgical LEDD controls	Presurgical LEDD SD controls	Postsurgical LEDD controls	Postsurgical LEDD SD controls
**1**										
**2**							1286	655.6	722	563.2
**3**	960	611	940	611	20	594				
**4**	1366	294	295	106			1306	692	591.2	431.6
**5**	1091	207	193	108						
**6**	1089	317	293	196			1355	732	534	439
**7**	460	151	141.7	203.6			1001.1	482.5	316	400.6
**8**	1289	588	322	254			1243	601	350	369
**9**	1093.2	458.6	468	256.7			1093.5	391.5	612.3	323.1
**10**										
**11**										
**12**	1296.9	665.2	1024.8	755.3			1392.6	808	790.4	345.7
**13**	1317	803	731	803	586	495				
**14**	1143	540	997	540	146	510				
**15**										
**16**										

LEDD: levodopa equivalent daily dose
SD: standard deviation

©2019 Artusi CA et al. *JAMA Network Open.*

eTable 3. Case report data

ID study	Study	Year	Genes	Gene type	Target	Follow-up (months)	Presurgical UPDRS-III mutated	Postsurgical UPDRS-III mutated	Presurgical LEDD mutated	Postsurgical LEDD mutated
**1**	Capecci et al	2004	PARKIN (homozygous)	1	STN	12	45	7	900	308
**2**	Romito et al	2005	PARKIN (homozygous)	1	STN	18	61	35	500	280
**3**	Genc et al	2016	PARKIN (heterozygous)	2	STN		48	7	200	100
**4**	Nakahara et al	2014	PARKIN + PINK1	3	STN	8	86	33	1181	490
**5**	Breit et al	2010	LRRK2	4	STN	12	70	24	900	500
**6**	Stefani et al	2012	LRRK2	4	STN	3	27	8	850	360
**7**	Moro et al	2008	PINK1	5	STN	12	35.5	18.99		
**8**	Antonini et al	2012	SNCA	6	STN	12	22	12.5	1250	460

UPDRS-III: unified parkinson's disease rating scale part III
LEDD: levodopa equivalent daily dose

©2019 Artusi CA et al. *JAMA Network Open.*

eTable 4. Meta-analysis output

ID study	Gene	Number of studies	I2	Absolute Effect	Confidence interval (lower)	Confidence interval (upper)	p-value	Percent Improvement	Group	Method
**1**	1	4	72.6	24.13	12.37	35.89	<0.001	43	1	D-L
**1**	1			24.13	4.6	43.6	0.03		1	HKSJ
**2**	2	5	52	23.02	15.21	30.83	<0.001	46	1	D-L
**2**	2			23.02	12.2	33.8	0.004		1	HKSJ
**3**	3	3	91.9	20	4.54	35.46	0.01	49	1	D-L
**3**	3			20	-20.4	60.4	0.17		1	HKSJ
**4**	4	8	85.5	25.24	21.27	29.22	<0.001	53	1	D-L
**4**	4			25.24	19.5	31	<0.001		1	HKSJ
**1**	1	3	90,2	494.818	-18.141	1007.776	0.06	61	2	D-L
**1**	1			494.818	-577.1	1566.8	0.19		2	HKSJ
**2**	2	3	22	711.899	491.809	931.988	<0.001	61	2	D-L
**2**	2			711.899	227.3	1196.5	0.02		2	HKSJ
**3**	3	2	0	269.16	226.833	311.488	<0.001	22	2	D-L
**3**	3			269.16	27.4	510.9	0.04		2	HKSJ
**4**	4	7	78.2	681.76	544.4	819.12	<0.001	55	2	D-L
**4**	4			681.76	539.8	823.7	<0.001		2	HKSJ

D-L: DerSimonian-Laird method
HKSJ: Hartung-Knapp-Sidik-Jonkman method

©2019 Artusi CA et al. *JAMA Network Open.*

eTable 5. Motor levodopa response

ID study	Study	Unique study	Year	Study type	Genes	Gene type	Target	Genes studied	Presurgical UPDRS-III mutated OFF levodopa	Presurgical UPDRS-III SD mutated OFF levodopa	Presurgical UPDRS-III mutated ON levodopa	Presurgical UPDRS-III SD mutated ON levodopa	Number of controls	Presurgical UPDRS-III controls OFF levodopa	Presurgical UPDRS-III SD controls OFF levodopa	Presurgical UPDRS-III controls ON levodopa	Presurgical UPDRS-III SD controls ON levodopa
**1**	Moro et al (a)	1	2008	Cohort	PARKIN (homozygous only)	1	STN	6	49.2	13.4	21.3	4.4	68	48.7	13.1	18.6	10.2
**2**	Moro et al (b)		2008	Cohort	PARKIN (heterozygous only)	2	STN	5	50	9.1	14.3	10.4					
**3**	Angeli et al (a)	1	2013	Cohort	PARKIN (homozygous), GBA, LRRK2	1	STN, Gpi, Vim										
**4**	Romito et al	1	2005	Cohort	PARKIN (heterozygous only)	2	STN	4	57	10.55	22.7	8.4	31	59.7	11.3	24.5	14.2
**5**	Lohmann et al (a)	1	2008	Cohort	PARKIN (homozygous)	1	STN	7	55.4	17.3	14.5	10	39	51.9	18.3	11.2	8.5
**6**	Lohmann et al (b)		2008	Cohort	PARKIN (heterozygous)	2	STN	7	54.3	13.9	11.6	12.7					
**7**	Kim et al	1	2014	Cohort	PARKIN (homozygous)	1	STN	3	49.8	24.5	18.3	7.8	9	38.3	10.6	17.7	12.6
**8**	Schupbac	1	2007	Cohort	LRRK2	3	STN	9	41.4	12.4	8.2	4.6	60	43.4	17	10.5	9.2

©2019 Artusi CA et al. *JAMA Network Open.*

	h et al																
**9**	Greenbaum et al	1	2013	Case-Control	LRRK2	3	STN	13	42.5	11.8	19.5	13	26	43.3	12.3	23.6	13.2
**10**	Sayad et al (a)	1	2016	Cohort	LRRK2	3	STN	15	55.8	16.4	25	13.2	10	51.7	14.4	30.6	16.7
**11**	Sayad et al (b)		2016	Cohort	PARKIN (heterozygous)	2	STN	2	47.5	2.1	30	2.8					
**12**	Lythe et al	1	2017	Case-Control	GBA	4	STN, Gpi	17	52.4	13	18.4	14.9	17	40.5	12	12.6	7.4
**13**	Angeli et al (b)		2013	Cohort	PARKIN (homozygous), GBA, LRRK2	3	STN, Gpi, Vim										
**14**	Angeli et al (c)		2013	Cohort	PARKIN (homozygous), GBA, LRRK2	4	STN, Gpi, Vim										
**15**	Gomez-Esteban et al	1	2006	Case-control	LRRK2	3	STN	4	48.5	18.5	18	7.4					
**16**	Weiss et al	1	2012	Case-Control	GBA	4	STN	3	39.5	19.1	16.5	3.5	6	46.2	11.8	18.3	7.1

UPDRS-III: unified parkinson's disease rating scale part III
SD: standard deviation

©2019 Artusi CA et al. *JAMA Network Open.*

eTable 6. Heterozygous *PRKN* analysis

META-ANALYSES

Age at PD onset (years; mean ± SD)	Disease duration at DBS (years; mean ± SD)	UPDRS-III improvement after STN DBS [95%CI] (%)	p-value UPDRS-III	LEDD changes after STN DBS [95%CI] (%)	p-value LEDD
38.4 ± 6.8	14.0 ± 2.0	20.5 [-2.2 - 43.3] (41%)	0.077	-921.2 [652.8 - 1189.7] (76%)	< 0.001

VALIDATION ANALYSES

N	I2	Pooled Mean change	95%CI		p-value	Baseline	Percent Improvement
UPDRS-III - Validation analysis #1							
4	96.6%	21.2	-0.97	43.3	0.061	50.1	42
UPDRS-III - Validation analysis #2							
4	95.7%	21.1	-1.3	43.4	0.065	50.1	42
LEDD - Validation analysis #1							
2	55.1%	924.0	655.1	1192.8	<0.001	1214.4	76
LEDD - Validation analysis #2							
2	41.5%	925.2	656.2	1194.1	<0.001	1214.4	76

CASE REPORT - INTRA-INDIVIDUAL PATIENT ANALYSES

Number of case reports	Mean baseline values	Mean improvement	Percent improvement
Motor improvement (UPDRS-III score)			
1	48	41	85%
LEDD reduction (mg)			
1	200	100	50%

Proportion and type of mutations

Proportion of mutated patients (95% CI)	Type of mutation	Study
9% (5%-12%)	AG 202–203del; C1101T; G535A; ex1dupl	Romito et al. 2005[28]
	E207fsX235; A206_E207ins28aa; R256C; R402P; G179?	Moro et al. 2008[23]
	R256C (2 patients); ex6dupl; ex6del; A398T; ex7dupl; ex3del	Lohmann et al. 2008[25]
	c.1000C>T; c.337_376del; c.1310C>T	Angeli et al. 2013[24]
	NR (2 patients)	Kim et al. 2014[26]
	c.458C>G; c.1204C>T	Sayad et al. 2016[29]
	c.89G>A	Genc et al. 2016[30]

LEDD: Levodopa Equivalent Daily Dose; PD: Parkinson Disease; UPDRS: Unified Parkinson's Disease Rating Scale; CI: confidence interval

Motor improvement was defined as the change in the UPDRS-III score between the presurgical Medication-OFF condition and the postsurgical Medication-OFF/Stimulation-ON condition.

©2019 Artusi CA et al. *JAMA Network Open.*

In the validation analyses, meta-analyses using random effects model with DerSimonian & Laird method were conducted for each outcome using the pooled standard deviation, computed after estimating the correlation coefficient between pre and post values for all datasets (Validation analysis #1) and ignoring the correlation between pre and post values (Validation analysis #2).

©2019 Artusi CA et al. *JAMA Network Open.*

eTable 7. Validation analyses for UPDRS-III improvement and LEDD reduction

	N	I2	Pooled Mean change	95%CI		p-value	Baseline	Percent Improvement
UPDRS-III Validation analysis #1								
PRKN	4	68.3%	24.0	12.3	35.7	<0.001	56.5	42
LRRK2	5	46.4%	22.9	15.1	30.7	<0.001	49.9	46
GBA	3	88.5%	20.1	3.95	36.1	0.015	40.5	50
UPDRS-III Validation analysis #2								
PRKN	4	59.6%	23.8	12.3	35.2	<0.001	56.5	42
LRRK2	5	30.3%	22.5	15.0	30.1	<0.001	49.9	45
GBA	3	85.8%	20.1	3.7	36.5	0.016	40.5	50
LEDD Validation analysis #1								
PRKN	3	89.4%	495.0	-18.4	1008.5	0.059	813.7	61
LRRK2	3	24.2%	713.2	492.1	934.3	<0.001	1164.7	61
GBA	2	0.0%	187.2	-40.4	414.7	0.107	1214.2	15
LEDD Validation analysis #2								
PRKN	3	86.1%	496.1	-19.8	1011.9	0.059	813.7	61
LRRK2	3	4.4%	701.6	488.2	914.9	<0.001	1164.7	60

CI: confidence interval

In the validation analyses, meta-analyses using random effects model with DerSimonian & Laird method were conducted for each outcome using the pooled standard deviation, computed after estimating the correlation coefficient between pre and post values for all datasets (Validation analysis #1) and ignoring the correlation between pre and post values (Validation analysis #2).

eTable 8. Pre STN DBS motor outcome (UPDRS-III score) associated with levodopa - Meta-analysis

Genes	Number of studies	I2	Pooled Mean change	95%CI		p-value	Baseline	Percent Improvement
PRKN	3	0.0%	32.2	21.2	43.1	<0.001	51.6	62
LRRK2	4	67.1%	28.9	23.5	34.3	<0.001	46.6	62
GBA	2	32.8%	32.2	24.2	40.2	<0.001	50.3	64
Heterozygous PRKN	4	79.2%	31.3	17.9	44.7	<0.001	50.1	62
Controls	9	82.6%	29.1	24.8	33.3	<0.001	44.9	64

CI: confidence interval

©2019 Artusi CA et al. *JAMA Network Open.*

eTable 9. Pre STN DBS motor outcome (UPDRS-III score) associated with levodopa - Intra-individual patient analysis

Genes	Number of case reports	Mean change (standard deviation)	Percent improvement
PRKN	2	39.0 (1.4)	76
LRRK2	2	27.0 (16.9)	56
PINK1	1	19.5	55
SNCA	1	18.0	64
PRKN+PINK1	1	61.0	71
Heterozygous PRKN	1	41.0	85

eAppendix 1. Statistical codes

```
clear
************eTable 1 and Table 2 analysis********************************
import excel using  datafile.xlsx, sheet("Meta-analysis for proportion") ///
firstrow
foreach var in mutation Parkinho Parkinhe LRRK2 GBA {
metaprop `var' screened, random
}
***generating pooled standard error and meta-analysis using D-L and HKSJ methods for motor score***
clear
import excel using  datafile.xlsx, sheet("Meta-analysis data") ///
firstrow clear
foreach var in ms ledd {
gen meanchange_case_`var'= pre_`var'_case-post_`var'_case
forval i=1/4 {
corr pre_`var'_case post_`var'_case if genetype==`i'
gen r_`var'_case`i'=r(rho)
gen se_`var'_case`i'= sqrt((sd_pre_`var'_case^2 + sd_post_`var'_case^2 -
2*r_`var'_case`i'*sd_pre_`var'_case*sd_post_`var'_case)/no_genes_studied) if genetype==`i'
metan meanchange_case_`var' se_`var'_case`i' if  genetype==`i',   sortby(year) lcols(study year) effect("Mean Change")
random
}
}

replace meanchange_case_ledd=meanchangedleddcase if  id==3 | id==14 |id==13
replace se_ledd_case1=sdchangedleddcase/sqrt(no_genes_studied) if id==3 & genetype==1
replace se_ledd_case2=sdchangedleddcase/sqrt(no_genes_studied) if id==3 & genetype==1
replace se_ledd_case3=sdchangedleddcase/sqrt(no_genes_studied) if  id==13   & genetype==3
replace se_ledd_case4=sdchangedleddcase/sqrt(no_genes_studied) if id==14 & genetype==4

forval i=1/4 {
metan meanchange_case_ms se_ms_case`i' if genetype==`i' , random rfdist
replace _WT=. if _WT==0
gen ymean=r(ES)
gen  df=r(df)
gen  y=meanchange_case_`var'  if genetype==`i'
gen  y2= (y-ymean)^2 if genetype==`i'
gen  y2w= y2*_WT if genetype==`i'
sum y2w if genetype==`i'
matrix hksj= r(sum)
gen  hksj1=hksj[1,1]/100
gen  hksj2= sqrt(hksj1/df)
gen  ll=  ymean-invttail(df,0.025)*hksj2
gen  ul=  ymean+invttail(df,0.025)*hksj2
gen t=ymean/hksj2
gen  p=2*ttail(df, t)
sum ll  ul p  if genetype==`i'
drop ymean df y y2 y2w hksj1  hksj2 t ll ul p
}


forval i=1/4 {
metan meanchange_case_ledd se_ledd_case`i' if genetype==`i' & meanchange_case_ledd !=. , random
replace _WT=. if _WT==0
gen ymean=r(ES)
gen  df=r(df)
gen  y=meanchange_case_ledd  if genetype==`i'
gen  y2= (y-ymean)^2 if genetype==`i'
gen  y2w= y2*_WT if genetype==`i'
sum y2w if genetype==`i'
```

©2019 Artusi CA et al. *JAMA Network Open.*

```
matrix hksj= r(sum)
gen  hksj1=hksj[1,1]/100
gen  hksj2= sqrt(hksj1/df)
gen  ll=  ymean-invttail(df,0.025)*hksj2
gen  ul=  ymean+invttail(df,0.025)*hksj2
gen t=ymean/hksj2
gen  p=2*ttail(df, t)
sum ll  ul p  if genetype==`i'
drop ymean df y y2 y2w hksj1  hksj2 t ll ul p
}

**generating pooled standard error and meta-analysis using D-L and HKSJ methods for motor score and ledd in control**
foreach var in ms ledd {
gen meanchange_con_`var'=pre_`var'_con-post_`var'_con
corr pre_`var'_con post_`var'_con
gen r_`var'_con=r(rho)
gen se_`var'_con= sqrt((sd_pre_`var'_con^2 + sd_post_`var'_con^2 -
2*r_`var'_con*sd_pre_`var'_con*sd_post_`var'_con)/no_control)
metan meanchange_con_`var' se_`var'_con ,   sortby(year) lcols(study year) effect("Mean Change") random
}

foreach var in ms ledd {
metan meanchange_con_`var' se_`var'_con ,  random rfdist
replace _WT=. if _WT==0
gen ymean=r(ES)
gen  df=r(df)
gen  y=meanchange_con_`var'
gen  y2= (y-ymean)^2
gen  y2w= y2*_WT
sum y2w
matrix hksj= r(sum)
gen  hksj1=hksj[1,1]/100
gen  hksj2= sqrt(hksj1/df)
gen  ll=  ymean-invttail(df,0.025)*hksj2
gen  ul=  ymean+invttail(df,0.025)*hksj2
gen t=ymean/hksj2
gen  p=2*ttail(df, t)
sum ll  ul p
drop ymean df y y2 y2w hksj1  hksj2 t ll ul p
}


*************Validation analysis ********************
foreach var in ms ledd {
corr pre_`var'_case post_`var'_case
gen r_`var'_case_p=r(rho)
gen se_`var'_case_p= sqrt((sd_pre_`var'_case^2 + sd_post_`var'_case^2 -
2*r_`var'_case_p*sd_pre_`var'_case*sd_post_`var'_case)/no_genes_studied)
gen se_`var'_case_a= sqrt((sd_pre_`var'_case^2 + sd_post_`var'_case^2)/no_genes_studied)
metan meanchange_case_`var' se_`var'_case_p , by(genetype)  sortby(year) lcols(study year) effect("Mean Change")
random
metan meanchange_case_`var' se_`var'_case_a , by(genetype)  sortby(year) lcols(study year) effect("Mean Change")
random
}
replace meanchange_case_ledd=meanchangedleddcase if  id==3 | id==14 |id==13
replace se_ledd_case_p=sdchangedleddcase/sqrt(no_genes_studied) if id==3 & genetype==1
replace se_ledd_case_p=sdchangedleddcase/sqrt(no_genes_studied) if id==3 & genetype==1
replace se_ledd_case_p=sdchangedleddcase/sqrt(no_genes_studied) if  id==13   & genetype==3
replace se_ledd_case_p=sdchangedleddcase/sqrt(no_genes_studied) if id==14 & genetype==4
replace meanchange_case_ledd=meanchangedleddcase if  id==3 | id==14 |id==13
replace se_ledd_case_a=sdchangedleddcase/sqrt(no_genes_studied) if id==3 & genetype==1
```

```
replace se_ledd_case_a=sdchangedleddcase/sqrt(no_genes_studied) if id==3 & genetype==1
replace se_ledd_case_a=sdchangedleddcase/sqrt(no_genes_studied) if  id==13   & genetype==3
replace se_ledd_case_a=sdchangedleddcase/sqrt(no_genes_studied) if id==14 & genetype==4

metan meanchange_case_ledd se_ledd_case_p , by(genetype)  sortby(year) lcols(study year) effect("Mean Change")
random
metan meanchange_case_ledd se_ledd_case_a , by(genetype)  sortby(year) lcols(study year) effect("Mean Change")
random


*******Meta-analysis for pre-scores for computing percent relative change*********************
foreach var in ms ledd {
gen se_pre_`var'_case =sd_pre_`var'_case/sqrt(no_genes_studied)
metan pre_`var'_case se_pre_`var'_case , by(genetype)  sortby(year) lcols(study year) effect("Mean Change") random
}
***************************Forest Plot Graph (Figure 2)****************
clear
import excel using  datafile.xlsx , sheet("graph") ///
firstrow
format I2 %9.1fc
label variable I2 "I-square(%)"
label define Gene1 1 "PRKN" 2 "LRRK2" 3 "GBA" 4 "Control"
label values Gene Gene1
admetan AbsoluteEffect LL UL if Group==1 , study(Method) by(Gene) sortby(Method) nosubgroup nowt  nooverall ///
  rcols( N I2 pvalue  Improvement) effect("Mean Change")  forestplot (xlabel (-65 -45 -25  0 25 45 65)  title("Pre-Post
Mean Changes in UPDRS-III", position(6) size(moderate) ) scheme(s1color) ///
  boxopts( msymbol(0) mcolor(white) ))
  graph export " datafile.xlsx \Figure2UPDRSf.wmf", replace

 admetan AbsoluteEffect LL UL if Group==2 , study(Method) by(Gene) sortby(Method) nosubgroup nowt  nooverall ///
  rcols( N I2 pvalue  Improvement) effect("Mean Change")  forestplot (xlabel (-1800 -1200 -600  0 600 1200 1800)
title("Pre-Post Mean Changes in LEDD", position(6) size(moderate) ) scheme(s1color) ///
  boxopts( msymbol(0) mcolor(white) ))
  graph export  “datafile.xlsx \Figure2leddf.wmf", replace

  ********* meta-analysis using D-L for motor score (pre-surgery)*******************
clear
import excel using datafile.xlsx, sheet("Levodopa ") ///
firstrow clear
foreach var in ms  {
gen meanchange_case_`var'= pre_`var'_case-post_`var'_case
forval i=1/4 {
corr pre_`var'_case post_`var'_case if genetype==`i'
gen r_`var'_case`i'=r(rho)
gen se_`var'_case`i'= sqrt((sd_pre_`var'_case^2 + sd_post_`var'_case^2 -
2*r_`var'_case`i'*sd_pre_`var'_case*sd_post_`var'_case)/no_genes_studied) if genetype==`i'
metan meanchange_case_`var' se_`var'_case`i' if  genetype==`i',   sortby(year) lcols(study year) effect("Mean Change")
random
}
}
 foreach var in ms  {
gen meanchange_con_`var'=pre_`var'_con-post_`var'_con
corr pre_`var'_con post_`var'_con
gen r_`var'_con=r(rho)
gen se_`var'_con= sqrt((sd_pre_`var'_con^2 + sd_post_`var'_con^2 -
2*r_`var'_con*sd_pre_`var'_con*sd_post_`var'_con)/no_control)
metan meanchange_con_`var' se_`var'_con ,   sortby(year) lcols(study year) effect("Mean Change") random
}

foreach var in ms  {
gen se_pre_`var'_case =sd_pre_`var'_case/sqrt(no_genes_studied)
metan pre_`var'_case se_pre_`var'_case , by(genetype)  sortby(year) lcols(study year) effect("Mean Change") random
```

©2019 Artusi CA et al. *JAMA Network Open.*

```
gen se_pre_`var'_con =sd_pre_`var'_con/sqrt(no_genes_studied)
metan pre_`var'_con se_pre_`var'_con ,   sortby(year) lcols(study year) effect("Mean Change") random
}

*******************Analysis of case reports******************************
clear
import excel using  datafile.xlsx, sheet("case-report data") ///
firstrow clear

foreach var in ms ledd  {
gen change_case_`var'= pre_`var'_case-post_`var'_case
gen pchange_case_`var'= (pre_`var'_case-post_`var'_case)/pre_`var'_case
}
gen change_ms_lev= pre_ms_lev-post_ms_lev
gen pchange_ms_lev= (pre_ms_lev-post_ms_lev)/pre_ms_lev
tabstat change_case_ms change_case_ledd pchange_case_ms pchange_case_ledd  change_ms_lev pchange_ms_lev,
by(genetype) stats (mean sd)
```

©2019 Artusi CA et al. *JAMA Network Open.*

eAppendix 2. Methods of data analysis

1. The data collection form provides the list of variables and their labels.
2. The excel datasheet (sheet 1: data for conducting meta-analysis of proportions, sheet 2 data for conducting meta-analysis of different outcomes, sheet 3: data for conducting analysis of case reports, sheet 4: data for producing forest plots) includes all data extracted from each article.
3. From each study, number of specific genes and total number of patients screened for any genes were extracted and included in excel datasheet 1. This database was used for estimating proportion of each specific gene mutation using meta-analysis. The statistical codes are also provided for conducting meta-analysis for estimating pooled proportions (see statistical codes file).
4. Wherever the study reported mean change and standard deviation of mean change, we used reported mean change and standard deviation of mean change in meta-analysis instead of pooled standard deviation obtained from standard deviation of each group.
5. Pearson's correlation coefficients between pre and post-intervention scores were computed for each outcome score separately for each gene (r_k where k is the specific gene group). The pooled standard deviation (PSD) was computed using standard deviation for pre-intervention score (SD_1), standard deviation for post-intervention score (SD_2) and r_k with formula $\sqrt{SD_1^2 + SD_2^2 - 2 * r_k * SD_1 * SD_2}$, and pooled standard error (PSE) was obtained using $\sqrt{SD_1^2 + SD_2^2 - 2 * r_k * SD_1 * SD_2/n}$, where is n is the number studies.
6. In the validation meta-analysis 1, the PSD was computed using $\sqrt{SD_1^2 + SD_2^2 - 2 * r * SD_1 * SD_2}$, and pooled standard error (PSE) was obtained using $\sqrt{SD_1^2 + SD_2^2 - 2 * r * SD_1 * SD_2/n}$, where r is the Pearson's correlation coefficient between pre and post-intervention scores irrespective of gene mutations. In the validation meta-analysis 2, the PSD was computed using $\sqrt{SD_1^2 + SD_2^2}$, and pooled standard error (PSE) was obtained using $\sqrt{SD_1^2 + SD_2^2/n}$.
7. Number of screened patients and number of specific genes were used to compute pooled proportion of specific gene mutation while mean change in outcome score along with PSE was used to compute pooled mean change in each outcome. In addition, meta-analysis was conducted to estimate the pooled pre-intervention score for each outcome to obtain percent relative change in each outcome after intervention separately for each gene. The DerSimonian-Laird (D-L) method of meta-analysis of proportions and baseline outcome scores were carried out while Hartung-Knapp-Sidik-Jonkman (HKSJ) method for random effects meta-analysis was conducted for outcome scores. In addition, D-L method was also applied for estimating pooled effect of intervention on each outcome for each specific gene separately. In validation meta-analysis for outcome scores, D-L method was used to compute pooled effects after estimating the correlation coefficient between pre and post values for all datasets (irrespective of gene mutations) and ignoring the correlation between pre and post values. We reported the number of studies included in each analysis, I^2 statistic value, pooled mean change or proportion, 95% confidence interval, p-value, and percent relative change from each outcome analysis.

©2019 Artusi CA et al. *JAMA Network Open.*
